# Neoadjuvant radiochemotherapy with cisplatin/5-flourouracil or carboplatin/paclitaxel in patients with resectable cancer of the esophagus and the gastroesophageal junction — comparison of postoperative mortality and complications, toxicity, and pathological tumor response

**DOI:** 10.1007/s00423-023-03091-0

**Published:** 2023-11-08

**Authors:** Eric Lorenz, Anna Weitz, Therese Reinstaller, Peter Hass, Roland S. Croner, Frank Benedix

**Affiliations:** 1 Department of General, Abdominal, Vascular and Transplant Surgery, University Hospital Magdeburg, Magdeburg, Germany; 2 Department of Radiation Therapy, Helios Hospital Erfurt, Erfurt, Germany

**Keywords:** Esophageal cancer, Esophagectomy, Radiochemotherapy, Complete response, Regression

## Abstract

**Purpose:**

In 2012, the CROSS trial implemented a new neoadjuvant radiochemotherapy protocol for patients with locally advanced, resectable cancer of the esophagus prior to scheduled surgery. There are only limited studies comparing the CROSS protocol with a PF-based (cisplatin/5-fluorouracil) nRCT protocol.

**Methods:**

In this retrospective, monocentric analysis, 134 patients suffering from esophageal cancer were included. Those patients received either PF-based nRCT (PF group) or nRCT according to the CROSS protocol (CROSS group) prior to elective en bloc esophagectomy. Perioperative mortality and morbidity, nRCT-related toxicity, and complete pathological regression were compared between both groups. Logistic regression analysis was performed in order to identify independent factors for pathological complete response (pCR).

**Results:**

Thirty-day/hospital mortality showed no significant differences between both groups. Postoperative complications ≥ grade 3 according to Clavien-Dindo classification were experienced in 58.8% (PF group) and 47.6% (CROSS group) (*p* = 0.2) respectively. nRCT-associated toxicity ≥ grade 3 was 30.8% (PF group) and 37.2% (CROSS group) (*p* = 0.6). There was no significant difference regarding the pCR rate between both groups (23.5% vs. 30.5%; *p* = 0.6). In multivariate analysis, SCC (OR 7.7; *p* < 0.01) and an initial grading of G1/G2 (OR 2.8; *p* = 0.03) were shown to be independent risk factors for higher rates of pCR.

**Conclusion:**

We conclude that both nRCT protocols are effective and safe. There were no significant differences regarding toxicity, pathological tumor response, and postoperative morbidity and mortality between both groups. Squamous cell carcinoma (SCC) and favorable preoperative tumor grading (G1 and G2) are independent predictors for higher pCR rate in multivariate analysis.

## Introduction

Each year, more than 450,000 patients are newly diagnosed with esophageal cancer (EC) worldwide [1]. The national cancer registries in Germany report about 10 cases per population of 100,000 annually [2]. Kamangar et al. reported similar rates throughout other countries in North America, Europe, and Oceania. However, especially in Asia, there are high-risk areas (e.g., China or Iran) where an incidence of up to 100 cases per 100,000 people annually was reported [3]. Histologically, there are two main types of esophageal cancer. Squamous cell carcinomas (SCC) are predominant worldwide but there is a growing incidence of adenocarcinomas (AC), especially in the western countries [4]. Despite huge efforts to improve diagnostic modalities and treatment options, long-term survival remains poor resulting in a 5-year survival of less than 40% [5].

In order to improve long-term survival, the standard multidisciplinary treatment for locally advanced but resectable esophageal cancer includes neoadjuvant radiochemotherapy (nRCT) or perioperative chemotherapy (nCT) followed by en bloc esophagectomy with radical lymph node dissection [6, 7]. There is growing evidence that nRCT protocols comprising chemotherapeutic agents such as carboplatin, cisplatin, paclitaxel, or 5-fluorouracil (5-FU) in combination with radiation doses of 40–50 Gray (Gy) are effective and improve the survival of patients without increasing perioperative morbidity or mortality [6, 8–10]. A complete pathological response (pCR), defined as the absence of viable tumor cells in the primary tumor and the resected lymph nodes, following nRCT, significantly increases 5-year survival [11, 12]. In 10–45% of the cases, nRCT achieved a complete pathological response (pCR) [11, 13, 14]. Thus, nRCT plays a key role in achieving R0 resection and pCR rates with an improved outcome for the affected patients. However, the optimal total radiation dosage and chemotherapy regime still remain unclear. In 2012, the CROSS trial yielded impressive survival results in patients receiving nRCT (carboplatin and paclitaxel with 41.4 Gy concurrent radiotherapy) prior to surgery compared to patients receiving surgery alone. nRCT resulted in a median overall postoperative survival of 49.4 months compared to 24.0 months [15]. This difference was even more impressive in patients suffering from SCC (81.6 months versus 21.1 months) [5].

A comparison of a PF-based (cisplatin/5-FU) neoadjuvant radiochemotherapy protocol (50.4 Gy) with the CROSS protocol showed significantly lower toxicity rates in the latter group while pathological response and long-term-survival were comparable [16]. Due to these results, we changed our local protocol used in patients with locally advanced, resectable esophageal and junctional cancer at the University Hospital Magdeburg. Since 2014, the CROSS protocol (carboplatin/paclitaxel + 41.4 Gy) is used in all patients with locally advanced, resectable esophageal and junctional carcinoma. However, recent publications have questioned the superiority of the CROSS protocol especially in patients suffering from squamous cell carcinoma (SCC). In these studies, lower rates of pCR and thus decreased survival were found when compared to PF-based nRCT protocols, warranting the need for periodic review of standardized nRCT protocols in EC [17].

The aim of this retrospective, unicentric study was to compare a cisplatin/5-FU-based nRCT protocol with the carboplatin/paclitaxel-based CROSS protocol regarding toxicity and adverse events during nRCT, pathological tumor response, and perioperative morbidity and mortality in patients suffering from locally advanced, resectable carcinoma of the esophagus or the gastroesophageal junction.

## Material and methods

### Patient selection

After obtaining Institutional Review Board approval, we retrospectively reviewed our prospectively maintained esophageal cancer database. In this study, we included all consecutive patients who received en bloc esophageal resection for locally advanced (uT3/T4, uN0-N3) adenocarcinoma or squamous cell carcinoma of the esophagus after nRCT at the University Hospital Magdeburg, Germany, between January 1, 2010, and March 31, 2022. Patients with junctional carcinoma (type II) were also considered for analysis if they had undergone nRCT prior to surgery. Patients with esophageal adenocarcinoma or junctional tumors who received perioperative chemotherapy were excluded from this study.

All patients in this study received neoadjuvant radiochemotherapy (nRCT) according to one of the following protocols: cisplatin/5-FU + 50.4 Gy (PF group) or carboplatin/paclitaxel + 41.4 Gy (CROSS group). Exclusion criteria were as follows:

- Patients who received perioperative chemotherapy
- Patients with recurrent cancer or distant metastasis
- Patients with synchronous carcinoma
- Patients with salvage esophagectomy for tumor recurrence/persistent tumor following definitive radiochemotherapy
- Patients who underwent transhiatally extended gastrectomy or reconstruction with colonic interposition

### Study parameters

Patient data were collected by reviewing medical records and the local esophageal cancer database. The following parameters were used for analysis:

- Demographics: age, gender, comorbidities, American Society of Anesthesiologists (ASA) score, body mass index (BMI, kg/m^2^),
- Type and severity of nRCT-associated toxicity and adverse events, completion of nRCT,
- Histopathological parameters: histological subtype, preoperative grading, resection margin status, postoperative nodal status, lymph node ratio, V and L status, presence of perineural infiltration, AJCC tumor stage [18],
- Evaluation of pathological tumor response:

- Squamous cell carcinoma — pathological tumor response was evaluated using the tumor regression grading system according to Mandard [19]
- Adenocarcinoma — pathological tumor response was evaluated using the regression grading system according to Becker [20]
- *Complete pathological tumor response (pCR)* was defined as the absence of viable tumor cells in the primary tumor and in the resected lymph nodes (ypT0 ypN0)
- *Major pathological tumor response* was defined as tumor regression grades 1–3 according to Mandard and tumor regression grades 1–2 according to Becker
- *Minor pathological tumor response* was defined as tumor regression grades 4–5 according to Mandard and tumor regression grade 3 according to Becker

- Operative parameters and length of ICU/hospital stay: surgical technique, operation time (minutes), intraoperative blood loss (ml), total length of hospital stay (days), total length of postoperative ICU stay (days),
- Postoperative complications according to the Clavien-Dindo classification [21]: general complications (pulmonary, cardiovascular, postoperative delirium); surgical complications (anastomotic leakage, chylothorax, persistent laryngeal nerve palsy, delayed gastric emptying, anastomotic stenosis, wound complication, 30-day mortality, in-hospital mortality).

### Neoadjuvant radiochemotherapy protocols

Patients of both cohorts were given a native planning-CT with a 3-mm slice thickness (Somatom Emotion 6, Siemens, Munich, Germany) in reproducible positioning according to institutional standards, provided that a complete staging including contrast-enhanced CT was available. The clinical target volume (CTV) was defined as the total tumor, including a cranio-caudal safety margin of 5 cm each and an axial safety margin of 1 cm. In addition, pathologically enlarged lymph nodes confirmed by endoluminal ultrasound were contoured, also dilated (1 cm safety margin), and then assigned to the CTV. All surrounding organs were contoured and digitized as risk structures (Organs At Risk = OAR) in the planning system Oncentra-Masterplan (Elekta, Stockholm, Sweden). A dosage calculation was then performed in order to be able to irradiate the CTV with the prescribed dosage, strictly taking into account the dose limits [22, 23] for the OAR. The prescription dosage for the classic nRCT protocol was 50.4 Gy for the PF group and 41.4 Gy for the CROSS group. The daily single dose for both groups was 1.8 Gy on 5 days per week. Patients treated according to the classic protocol received chemotherapy as suggested by the study of Herskovic with cisplatin (15 mg/m^2^/day) and 5-FU (1000 mg/m^2^/day) in week 1 (days 1–5) and week 5 (days 1–5) of concurrent radiation [24]. Patients treated according to the CROSS protocol received carboplatin (area under the curve = 2) and paclitaxel (50 mg/m^2^) on days 1, 8, 15, 22, and 29. In the case of relevant contraindications (e.g., renal insufficiency), cisplatin was replaced by carboplatin. nRCT-associated toxicity with corresponding severity was categorized using The National Cancer Institute Common Terminology Criteria for Adverse Events (CTCAE) Version 4.0 [25]. Early discontinuation of nRCT was defined as not receiving the full dosage of radiotherapy and chemotherapy, respectively, according to the initial nRCT protocol.

### Surgical resection

Ivor Lewis esophagectomy with two-field lymphadenectomy and a circular stapled intrathoracic anastomosis was performed in all patients with junctional tumors and esophageal carcinomas of the middle and lower third of the esophagus. McKeown esophagectomy was performed in patients with tumors of the upper third of the esophagus. For reconstruction, a 3-cm-wide gastric tube was used in all patients. Surgical techniques varied throughout the study period with open procedures used at the beginning. Later, we established the laparoscopic hybrid esophagectomy which was the preferred approach used between 2013 and 2017. In 2018, robotic-assisted minimally invasive esophagectomy (hybrid and full robotic approach) was introduced in our department and is currently the standard approach used in patients undergoing elective en bloc esophagectomy. Details of further technical aspects have been described in a recent publication [26].

### Statistical analysis

Statistical analysis was performed using the SPSS Version 24 software package (IBM Corporation, Armonk, NY, USA). In univariate analysis, categorical variables (nominal/ordinal) are given as absolute (*n*) and/or relative frequencies (%). Differences between the groups were tested using Pearson’s chi-squared test (*χ*^2^). Fisher’s exact test was alternatively used if applicable. Continuous variables are expressed as mean (SD — standard deviation) and range. Differences between continuous variables were tested using Student’s *t*-test or the Mann-Whitney *U* test depending on the scale level. No adjustment for multiple testing was applied in any of the analyses. A multiple imputation for missing values was not performed. Univariate logistic regression was performed in order to select useful predictors for complete pathological response and severe postoperative morbidity (≥ Clavien-Dindo 3). All predictors with a *p*-value < 0.01 were used in multivariate logistic regression. Calculated differences with a two-sided *p*-value < 0.05 were considered to be significant (no adjustment for multiplicity).

## Results

### Preoperative demographics, clinical, and histopathological characteristics (Table 1)

In total, 134 patients were considered for analysis. All of them underwent nRCT followed by en bloc esophagectomy during the study period. The mean age was 61 years ± 9.5. There was a predominance of male patients (88.1%). Fifty-two (38.8%) patients received cisplatin- and 5-fluorouracil-based nRCT with concurrent radiation at 50.4 Gy (PF group). Eighty-two (61.2%) patients received carboplatin- and paclitaxel-based nRCT in combination with a total radiation dosage of 41.1 Gy according to the CROSS protocol (CROSS group). The preoperative demographics and clinical characteristics are summarized in Table 1. There were no significant differences between both groups regarding gender, age, ASA score, or BMI. The preoperative tumor grading and the histological subtype also did not differ significantly between the groups. However, cardiovascular comorbidity was more frequently diagnosed in PF group (PF group: 59.6% vs. CROSS group: 41.5%; *p* = 0.04) (Table 1).

**Table 1:** Preoperative demographics, clinical, and histopathological characteristics

	Total cohort (*n* = 134)	PF group (*n* = 52)	CROSS group (*n* = 82)	*p*-value
Gender, *n* (%)				0.9
Male	118 (88.1)	46 (88.5)	72 (87.8)	
Age (years)				0.1
Mean ± SD	61.02 ± 9.5	62.6 ± 9.8	60.9 ± 9.3	
Range	39–82	44–79	39–82	
BMI (kg/m^2^)				0.2
Mean ± SD	24.7 ± 5.1	24.0 ± 5.0	25.2 ± 5.2	
Range	16.0–47.5	16.0–42.3	17.0–47.5	
ASA score, *n* (%)				0.2
1 + 2	80 (60.2)	34 (66.7)	46 (56.1)	
3 + 4	53 (39.8)	17 (33.3)	36 (43.9)	
Comorbidities, *n* (%)				
Diabetes mellitus	23 (17.2)	11 (21.2)	12 (14.6)	0.33
Pulmonary comorbidity	38 (28.4)	15 (28.8)	23 (28.0)	0.9
Cardiovascular comorbidity	65 (48.5)	31 (59.6)	34 (41.5)	0.04
Smoking	88 (66.2)	38 (73.1)	50 (61.7)	0.2
Histological subtype, *n* (%)				0.2
Squamous cell carcinoma (SCC)	98 (73.1)	35 (67.3)	63 (76.8)	
Adenocarcinoma	36 (26.9)	17 (32.7)	19 (23.2)	
Preoperative tumor grading, *n* (%)				0.1
G1 — well differentiated	4 (3.8)	0 (0 )	4 (3.8)	
G2 — moderately differentiated	73 (70.2)	34 (79.1)	39 (63.9)	
G3 — Poorly differentiated	27 (26.0)	9 (20.9)	18 (29.5)	

PF group: 50.4 Gy + 5 x Cisplatin/5-FU; CROSS group: 41.4 Gy + 2 x Carboplatin/Paclitaxel

*SD*, standard deviation; *BMI*, body mass index; *ASA*, American Society of Anesthesiologists

### Toxicity of neoadjuvant radiochemotherapy (Table 2)

The percentage of patients who fully completed their nRCT according to the protocol and the nRCT-associated toxicity are displayed in Table 2. nRCT was considered incomplete in all cases where the daily chemotherapy dosage was reduced or the chemotherapy was prematurely discontinued. By contrast, in cases where carboplatin was used instead of cisplatin due to relevant contraindications, nRCT was not considered incomplete provided that all chemotherapy cycles were applied. The toxicity with corresponding severity during nRCT was categorized in accordance with The National Cancer Institute Common Terminology Criteria for Adverse Events (CTCAE) Version 4.0. All patients in this study received the full radiation dosage according to the individual protocol. In the PF group, 4 (7.7%) patients received carboplatin instead of cisplatin due to side effects or preexisting comorbidities (data not shown). Severe hematological and non-hematological toxicity ≥ grade 3 were not significantly different between both groups. However, there was a higher rate of leukopenia in the CROSS group resulting in premature discontinuation of chemotherapy. Therefore, the percentage of patients who fully completed nRCT was significantly higher in the PF group (PF group: 96.2% vs. CROSS group: 71.3%; *p* < 0.01) (Table 2).

**Table 2:** Completion and toxicity of neoadjuvant radiochemotherapy according to protocol

	Total cohort (*n* = 134)	PF group (*n* = 52)	CROSS group (*n* = 82)	*p*-value
Completion of nRCT, *n* (%)	107 (81.1)	50 (96.2)	57 (71.3)	<0.01
Overall toxicity ≥ grade 3, *n* (%)	45 (34.6)	16 (30.8)	29 (37.2)	0.6
Hematological toxicity ≥ grade 3, *n* (%)	26 (20.0)	10 (19.2)	16 (20.5)	1.0
Anemia	2 (7.7)	1 (10.0)	1 (6.3)	
Leukopenia	17 (65.4)	5 (50.0)	12 (75.0)	
Thrombocytopenia	4 (15.4)	2 (20.0)	2 (12.5)	
Thrombosis	3 (11.5)	2 (20.0)	1 (6.3)	
Non-hematological toxicity ≥ grade 3, *n* (%)	19 (14.2)	7 (13.5)	12 (14.6)	0.8
Nausea	2 (10.5)	0 (0)	2 (16.7)	
Esophagitis	16 (84.2)	6 (85.7)	10 (83.3)	
Dermal irritation	1 (5.3)	1 (14.3)	0 (0)	

PF group: 50.4 Gy + 5 x Cisplatin/5-FU; CROSS group: 41.4 Gy + 2 x Carboplatin/Paclitaxel

### Histopathological parameters and pathological tumor response (Table 3)

Negative resection margins could be achieved in 90.2% (PF group) and 95.1% (CROSS group) of the patients, respectively, without any significant difference between both groups (*p* = 0.3). The number of lymph nodes retrieved was significantly higher in the CROSS group (20 vs. 25; *p* = 0.003). However, there was no statistically significant difference regarding the mean number of lymph nodes involved (PF group: 1.4 vs. CROSS group: 1.2; *p* = 0.7) or the mean lymph node ratio (PF group: 0.08 vs. CROSS group: 0.05; *p* = 0.3) (Table 3).

**Table 3:** Histopathological parameters and tumor regression

	Total cohort (*n* = 134)	PF group (*n* = 52)	CROSS group (*n* = 82)	*p*-value
Resection margin status, *n* (%)				
R0	124 (93.2)	46 (90.2)	78 (95.1)	0.3
Number of LN retrieved				
Mean ± SD	22.9 ± 9.9	19.9 ± 7.7	24.7 ± 10.7	0.003
Range	4–68	6–46	4–68	
Number of LN involved				0.7
Mean ± SD	1.27 ± 2.3	1.37 ± 2.6	1.21 ± 2.1	
Range	0–12	0–12	0–10	
Mean LN ratio	0.065 ± 0.14	0.084 ± 0.18	0.053 ± 0.1	0.3
V status (V1)	14 (10.9)	3 (6.1)	11 (13.8)	0.2
L status (L1)	37 (28.5)	11 (22.0)	26 (32.5)	0.2
Perineural infiltration (Pn1)	32 (24.8)	9 (18.0)	23 (29.1)	0.2
Postoperative tumor stage (AJCC 8th Ed.)				0.8
0	37 (27.8)	12 (23.5)	25 (30.5)	
1	29 (21.8)	12 (23.5)	17 (20.7)	
2	12 (9.0)	6 (11.8)	6 (7.3)	
3	48 (36.1)	18 (35.3)	30 (36.6)	
4	7 (5.3)	3 (5.9)	4 (4.9)	
Pathological tumor response				
Complete response, *n* (%)	37 (27.8)	12 (23.5)	25 (30.5)	0.6

PF group: 50.4 Gy + 5 x Cisplatin/5-FU; CROSS group: 41.4 Gy + 2 x Carboplatin/Paclitaxel

*LN*, lymph node; *LN ratio*, number of LN involved/number of LN retrieved; *AJCC 8th Ed.*, American Joint Committee on Cancer, 8th ediiton

There was also no significant difference between both groups regarding the postoperative AJCC tumor stage, V status, L status, and Pn status (*p* = 0.8; *p* = 0.2; *p* = 0.2; *p* = 0.2). A complete pathological response (pCR) was more frequently observed in the CROSS group (PF group: 23.5% vs. CROSS group: 30.5%). However, this difference was not statistically significant (*p* = 0.6) (Table 3).

For the evaluation of complete pathological tumor response, a subgroup analysis for both histological subtypes was also performed. Like in the analysis of the entire cohort, no significant differences could be demonstrated between the PF group and CROSS group for both histopathological subtypes separately (SCC: *p* = 0.7; adenocarcinoma: *p* = 0.7). However, patients with SCC showed significantly higher rates of complete pathological response compared to those with adenocarcinoma especially in the CROSS group (38.1% vs. 5.3%; *p* < 0.01) (Table 3).

### Operative parameters and postoperative course (Table 4)

Operative parameters are summarized in Table 4. The type of surgical resection was not significantly different between both groups. However, minimally invasive procedures including conventional and robotic approaches were less frequently performed in the PF group (15.4% vs. 26.8%; 0% vs. 51.2%; *p* ≤ 0.01). By contrast, the mean operative time was significantly longer in the CROSS group (305 min vs. 394 min; *p* < 0.01). The mean hospital length of stay and the mean total ICU length of stay were not significantly different between both groups (*p* = 0.6; *p* = 0.1) (Table 4).

**Table 4:** Operative parameters and postoperative course

	Total cohort (*n* = 134)	PF group (*n* = 52)	CROSS group (*n* = 82)	*p*-value
Type of resection, *n* (%)				0.2
Ivor Lewis esophagectomy	105 (78.4)	44 (84.6)	61 (74.4)	
McKeown esophagectomy	29 (21.6)	8 (15.4)	21 (25.6)	
Surgical approach, *n* (%)				<0.01
Open	62 (46.3)	44 (84.6)	18 (22.0)	
Conventional minimally invasive	30 (22.4)	8 (15.4)	22 (26.8)	
Robot-assisted	42 (31.3)	0 (0)	42 (51.2)	
Operative time (min)				<0.01
Mean ± SD	359.7 ± 83.3	305.2 ± 68.7	394.2 ± 72.8	
Range	185.0 ± 659.0	185.0 ± 490.0	231.0 ± 659.0	
Hospital length of stay (days)				0.6
Mean ± SD	25.7 ± 15.4	26.6 ± 14.2	25.1 ± 16.2	
Range	8–77	11–71	8–77	
Po ICU stay (days)				0.08
Mean ± SD	7.8 ± 10.8	6.0 ± 6.2	8.9 ± 12.9	
Range	1–74	1–30	1–74	
Total ICU stay (days)				0.1
Mean ± SD	10.3 ± 13.5	8.1 ± 9.7	11.6 ± 15.4	
Range	1–74	1–45	1–74	

PF group: 50.4 Gy + 5 x Cisplatin/5-FU; CROSS group: 41.4 Gy + 2 x Carboplatin/Paclitaxel

*po* postoperative, *ICU* intensive care unit

### Postoperative morbidity and mortality (Table 5)

Postoperative morbidity and mortality are summarized in Table 5. There were no statistically significant differences between both groups for any of the postoperative general and surgical complications analyzed in this study. This was also confirmed for complications ≥ grade 3 according to Clavien-Dindo classification (*p* = 0.2). Likewise, mortality did not differ significantly between the groups (30-day mortality: *p* = 0.2; hospital mortality: *p* = 0.3) (Table 5).

**Table 5:** Postoperative morbidity and mortality

	Total cohort (*n* = 134)	PF group (*n* = 52)	CROSS group (*n* = 82)	*p*-value
30-day mortality, *n* (%)	2 (1.5)	2 (3.8)	0 (0)	0.2
Hospital mortality, *n* (%)	8 (6.0)	5 (9.6)	3 (3.7)	0.3
Complication Clavien-Dindo ≥ grade 3	69 (51.9)	30 (58.8)	39 (47.6)	0.2
Surgical complication				
Anastomotic leakage	27 (20.1)	13 (25.0)	14 (17.8)	0.7
Chylothorax	26 (19.4)	9 (17.3)	17 (20.7)	0.7
Laryngeal nerve palsy	14 (10.5)	4 (7.7)	10 (12.3)	0.4
Anastomotic stenosis	6 (4.5)	3 (5.8)	3 (3.7)	0.7
Delayed gastric emptying	22 (16.7)	12 (23.1)	10 (12.5)	0.2
Wound complication	11 (8.2)	4 (7.7)	7 (8.5)	1.0
General complication				
Pulmonary	38 (28.4)	14 (26.9)	24 (29.3)	0.8
Cardiovascular	4 (3.0)	2 (3.8)	2 (2.4)	0.6
Postoperative delirium	28 (20.9)	10 (19.2)	18 (22.0)	0.8

PF group: 50.4 Gy + 5 x Cisplatin/5-FU; CROSS group: 41.4 Gy + 2 x Carboplatin/Paclitaxel

*PO* postoperative, *ICU* intesive care unit

^1^Severe complication ≥ Clavien-Dindo grade 3

### Univariate and multivariate analyses of independent predictors associated with complete pathological tumor response (pCR) (Table 6)

The results of our univariate and multivariate logistic regression analyses of independent predictors for complete pathological tumor response (pCR) are displayed in Table 6. In univariate analysis, three parameters were significantly associated with pCR: histological subtype (*p* = 0.003), preoperative tumor grading (*p* = 0.02), and smoking (*p* = 0.04).

**Table 6:** Univariate and multivariate logistic regression analyses of independent predictors associated with complete pathological tumor response (pCR) after nRCT followed by en bloc esophagectomy in patients suffering from esophageal/junctional cancer

Parameter	Univariate analysis			Multivariate analysis		
	Odds ratio	95% CI	*p*-value	Odds ratio	95% CI	*p*-value
Histological subtype						
Adenocarcinoma (reference)						
Squamous cell carcinoma	9.32	2.11–41.17	< 0.01	7.70	1.70–34.77	< 0.01
Preoperative tumor grading						
G3 (reference)						
G1/G2	2.64	1.15–6.06	0.02	2.79	1.14–6.83	0.03
Smoking						
Smoking (reference)						
Non-smoking	0.39	0.15–0.95	0.04	0.44	0.16–1.16	0.09

*CI* confidence interval

Multivariate analysis confirmed this association for the histological subtype (*p* < 0.01) and for preoperative tumor grading (*p* = 0.03). In both analyses, no significant influence of the type of nRCT protocol was observed (data not shown).

### Subgroup analysis of outcome variables in patients suffering from squamous cell carcinoma (SCC) vs. adenocarcinoma (AC) (Table 7)

Relevant outcome variables dependent of histological subtype are displayed in Table 7. There were no statistically significant differences for postoperative complications ≥ grade 3 according to Clavien-Dindo classification between SCC and AC (*p* = 0.6). Likewise, mortality did not differ significantly between both groups (30-day mortality: *p* = 0.6; hospital mortality: *p* = 0.4). Completion rate of nRCT was comparable between groups (*p* = 0.6). However, pathological complete response following nRCT was significantly higher in SCC compared to AC (35.1% vs. 5.6%; *p* < 0.001).

**Table 7:** Subgroup analysis of outcome variables in patients suffering from squamous cell carcinoma (SCC) vs. adenocarcinoma (AC) of the esophageus

	Total cohort (*n* = 134)	SCC (*n* = 98)	AC (*n* = 36)	*p*-value
Completion of nRCT, *n*/valid cohort size (%)	107 (81.1)	78 (79.6)	29 (85.3)	0.6
Complication Clavien-Dindo ≥ grade 3	69 (51.9)	50 (51.5)	19 (52.8)	1.0
30-day mortality, *n* (%)	2 (1.5)	1 (1.0)	1 (2.8)	0.6
Hospital mortality, *n* (%)	8 (6.0)	5 (5.1)	3 (8.3)	0.4
Pathological complete response, *n* (%)	37 (27.8)	35 (35.1)	2 (5.6)	<0.001
- Following PF-nRCTx		11/34 (32.4)	1/17 (5.9)	0.08
- Following CROSS-RCTx		24/64 (24.5)	1/19 (5.3)	0.003

*SCC* squamous cell carcinoma, *AC* adenocarcinoma

PF: 50.4 Gy + 5 x Cisplatin/5-FU; CROSS: 41.4 Gy + 2 x Carboplatin/Paclitaxel

## Discussion

Neoadjuvant radiochemotherapy has been proven beneficial in patients with locally advanced, resectable esophageal and junctional cancer prior to surgery. Advantages of nRCT include downstaging of the tumor, a higher rate of negative resection margins, and an improved overall survival [15, 27, 28]. A meta-analysis showed that postoperative morbidity was not significantly increased in patients who received nRCT prior to surgery when compared to patients who underwent surgery alone [29].

However, the currently used nRCT protocols vary significantly regarding the type and dosage of chemotherapeutic agents (i.e., cisplatin, carboplatin, 5-fluorouracil, paclitaxel, irinotecan) as well as the radiation dosage [5, 16, 27, 29, 30]. In this study, we compared two patient cohorts who received trimodal therapy with either one of two nRCT protocols (cisplatin/5-fluorouracil + 50.4 Gy vs. carboplatin/paclitaxel + 41.4 Gy) prior to elective en bloc esophagectomy. We found no significant differences between both groups regarding RCT-associated toxicity, R0 resection rates, and complete or major pathological tumor response. Mortality rates (10% vs. 4%) and postoperative complications ≥ grade 3 according to Clavien-Dindo classification (59% vs. 48%) were lower in the CROSS group but without the differences being significant.

The overall completion rate of nRCT as per protocol was 81% for the entire cohort. However, significantly less patients in the CROSS group completed all cycles of chemotherapy (96% vs. 57%; *p* < 0.01). Likewise, severe leukopenia exceeding grade 3 toxicity was more common in the CROSS group. However, this difference was not statistically significant. In our cohort, there were no significant differences regarding the incidence of hematological or non-hematological side effects. Sanford et al. also found no significant differences in neutropenic fewer among different nRCT protocols including cisplatin/5-FU (11.4%), cisplatin/irinotecan (6.5%), and carboplatin/Taxol (5.2%). For non-hematological side effects, however, this study found a significantly higher incidence of diarrhea (0% vs. 1.3%; *p* = 0.007) and esophagitis (5.7% vs. 18.2%; *p* = 0.005) in the CROSS group. All other side effects were comparable [31]. These results are in line with those published by Münch et al. who also compared two nRCT protocols (CROSS protocol vs. protocol based on cisplatin/5-fluorouracil + 45 Gy) and found that nearly 50% of all patients in both groups developed severe myelotoxic side effects exceeding grade 3 but without significant difference between both groups [32].

Previous studies also suggested that taxane-based chemotherapy might increase the risk of myelosuppression and febrile neutropenia when compared to 5-FU-based chemotherapy (RR 1.28; *p* = 0.02; grade 3+4 toxicity: 19.5% vs. 69%; *p* < 0.001) [33, 34]. These results differ from those reported by Honing et al. who compared carboplatin/paclitaxel vs. cisplatin/5-fluorouracil as chemotherapy agents used in two different definitive radiochemotherapy protocols [30]. They found a significant advantage for the carboplatin/paclitaxel-based protocol with fewer severe side effects (22% vs. 55%) and, consecutively, a higher completion rate of the definitive RCT (82% vs. 57%). Other authors also support the hypothesis that patients treated according to a cisplatin/5-fluorouracil-based RCT protocol suffer significantly more often from severe overall/hematological/non-hematological side effects exceeding grade 3 (common terminology for adverse events — CTCAE) when compared to the CROSS protocol [16]. Presumably, the different results regarding RCT-associated toxicity in many studies can be attributed to the heterogeneity of protocols including different agents and dosages used for concurrent chemotherapy, and different total radiation dosages and application periods.

In our analysis, pCR was achieved in 24% of the patients in the PF group and in 31% of the cases who received nRCT according to the CROSS protocol. However, this difference was without statistical significance. Our observed percentage of pCR is in line with the pCR stated in the original publication by Hagen et al. with a total pCR rate of 29% in their cohort [15]. Our results are also in line with prior studies that showed that complete pathologic response rates following PF-based nRCT vary between 13 and 45% compared to 25% and 30% following paclitaxel-based nRCT [13, 16, 32]. In contrast to our results, Haisley et al. found that the cisplatin/5-fluorouracil-based nRCT protocol resulted in a 2.7-fold increased pCR rate when compared to the carboplatin/paclitaxel-based nRCT protocol (*p* = 0.03). Furthermore, they were able to show an improved recurrence-free survival (hazard ratio: 0.39, *p* < 0.01) and overall survival (hazard ratio: 0.46; *p* < 0.05) [35]. Similarly, Sanford et al. showed that the cisplatin/5-fluorouracil-based nRCT protocol is associated with higher rates of major or complete pathological response compared to the carboplatin/paclitaxel-based nRCT protocol (37% vs. 17%; *p* = 0.02) [31].

In a subgroup analysis, 35.1% of all SCC tumors showed pCR compared to 5.6% pCR in all AC tumors (*p* < 0.001) following nRCTx independently of protocol used (Table 7). These results are in line with the results of other studies. In the original CROSS trial, pCR was observed in 23% of AC and 49% of SCC patients (*p* = 0.008). Likewise, the study by Sanford et al. found a significantly higher rate of pCR among SCC patients compared to AC patients (60.7% vs. 18.4%; *p* < 0.001) following the CROSS protocol [31]. However, in contrast to these studies, the total rate of pCR in AC and SCC patients was lower in our cohort.

When comparing both nRCT protocols, we found a higher rate of pCR in the CROSS group (30.5 % vs. 23.5 % in the PF group). However, this difference was not statistically significant. These results are in line with those reported by Wong et al. in 2020. In their study, they found a pCR rate of 24.6% for the cisplatin/5-fluorouracil-based nRCT protocol and 35.5% for the CROSS protocol. Like in our study, no significant difference between both nRCT protocols was observed (*p* = 0.15) [17]. By contrast, Gao et al. showed a significantly higher rate of pCR in SCC patients in the PF group compared to the CROSS protocol (29.5% vs. 45.3%; *p* = 0.002) [13].

In a multivariate analysis, we then investigated parameters that significantly influence the pCR rate. We found that patients suffering from SCC had a 7.7-fold higher likelihood of pCR irrespective of the nRCT protocol used. These results are in line with those published by Worrell et al. in 2020. They found that AC was associated with a 0.5-fold decreased likelihood of pCR compared to SCC (*p* < 0.001) [36]. Furthermore, Donahue et al. also confirmed that SCC was associated with significantly higher rates of pCR compared to AC [11]. By contrast, Sanford et al. showed that only the prevalence of signet cells in AC was associated with a 0.78-fold decreased pCR [37]. Likewise, Nehlsen et al. failed to show a significant influence of the histological subtype on pCR in their study [38].

In addition, we found that a favorable initial tumor grading (G1/G2) was associated with a 2.6-fold increased likelihood of pCR. These results are confirmed by the findings of Blum et al. published in 2017. They found that poor tumor grading (G3) was associated with a 0.4-fold decreased likelihood of pCR (*p* < 0.001) [37].

There is some evidence that female gender may also influence the pCR rate. Samson et al. reported a 4% increased complete pathological response rate (*p* = 0.004) in female patients suffering from esophageal cancer [12]. These results are in line with the findings of a study by Donahue et al. published in 2009 [11]. They found that male gender was associated with a 0.15-fold reduced probability of pCR [36]. However, our study did not confirm any significant influence of gender on the pCR rate. This is in line with the results of Nehlsen et al. published in 2021. In their study, no significant association between gender and the pCR rate was observed (*p* = 0.25) [38]. Several studies were able to show that pCR is significantly associated with an improved median overall survival (60 months vs. 30 months: *p* < 0.001) and an improved 5-year survival rate (55% vs. 26%; *p* = 0.01) [11, 12]. However, even today, no reliable markers or diagnostic tools are available to safely predict complete tumor regression following nRCT prior to surgical intervention [39]. Unnecessary surgery could be avoided in these patients. With the aim to clarify this issue, two randomized studies are currently evaluating active surveillance concepts in patients with esophageal cancer and complete tumor response after nRCT. For the SANO trial, first results are expected to be published in late 2023 [40]. The ESOSTRATE trial is still recruiting patients; results will possibly be available in 2025 [41].

In our study, postoperative morbidity or mortality did not differ significantly between both nRCT protocols. Most of the general complications were pulmonary complications. 28.4% of the patients we studied developed a pulmonary complication, without any significant difference between both groups. These results are in line with those published by Blom et al. in 2014. They reported rates of pulmonary complications of 33% and 34%, respectively, without any significant difference between the two nRCT protocols analyzed in their study (*p* = 0.9) [16]. By contrast, Gao et al. reported a significantly higher rate of pulmonary complications in the CROSS group (14.1%) when compared to an nRCT based on cisplatin/5-fluorouracil + 50.4 Gy (11.3%) (*p* < 0.01) [13]. In our study, the surgical complication rate also did not differ significantly between both groups. Like we found in our study, Blom et al. also reported no significant difference in leakage rates between a cisplatin/5-fluorouracil-based nRCT protocol and the CROSS protocol (*p* = 0.4). Wong et al. reported an anastomotic leakage rate of 15.9% in the CROSS group and 9.2% in the PF group, without any significant difference between both groups (*p* = 0.2) [16, 17]. By contrast, Gao et al. found a significant decrease in anastomotic leakage rates after introducing the CROSS protocol (27% vs. 10%; *p* < 0.001) [13].

Regarding postoperative mortality, our study did not find any statistically significant differences between both groups. This is confirmed by two other studies. Blom et al. stated an in-hospital mortality rate of 6% in the PF group and of 2% in the CROSS group (*p* = 0.25). Wong et al. also did not find any significant difference regarding in-hospital mortality between the two nRCT protocols analyzed (cisplatin/5-FU: 2.6% vs. CROSS protocol: 4.3%; *p* = 0.9) [16, 17].

There are a few limitations of our study that should be acknowledged. First, the small sample size and the unicentric retrospective design might have influenced the results. Second, our data was collected over a time period of 11 years with varying surgeons. This makes it susceptible to changes in surgical approach and experience as well as changes in postoperative management of the affected patients. For example, minimally invasive procedures including conventional and robotic approaches were less frequently performed in PF group. Third, recurrence-free and overall survival of the patients was not analyzed in this study. This would, however, be of paramount importance for a final assessment of the efficiency of the nRCT protocol. This issue will be addressed in a future study.

## Conclusion

The present study showed that both nRCT protocols are effective and safe. There were no significant differences regarding RCT-associated toxicity, pathological tumor response, and postoperative morbidity and mortality despite a lower RCT completion rate and a lower radiation dose in the CROSS group. Furthermore, the type of nRCT used had no significant influence on the pCR rate. Based on these results, we will continue using the CROSS protocol in our institution in patients with locally advanced esophageal and junctional cancer.

Further research is required in order to determine which patients may benefit from which type of nRCT taking into account their different risk profiles. In the future, a tailored approach with respect to the individual patient and their tumor characteristics is essential for improving multimodal therapy and thus overall survival.

## References

[1] Pennathur A, Gibson MK, Jobe BA, Luketich JD (2013). Oesophageal carcinoma. Lancet (London, England).

[2] Kaatsch P, Spix C, Katalinic A, Hentschel S, Luttmann S, Stegmaier C (2016) Cancer in Germany 2011/2012. Robert Koch Institute and the association of population-based cancer registries in Germany, Robert Koch Institute, Berlin https://www.krebsdaten.de/Krebs/EN/Content/Publications/Cancer_in_Germany/cancer_chapters_2011_2012/cancer_germany_2011_2012.pdf?__blob=publicationFile; Accessed 15 Jan 2023

[3] Kamangar F, Dores GM, Anderson WF (2006). Patterns of cancer incidence, mortality, and prevalence across five continents: defining priorities to reduce cancer disparities in different geographic regions of the world. J Clin Oncol.

[4] Melhado RE, Alderson D, Tucker O (2010). The changing face of esophageal cancer. Cancers..

[5] Shapiro J, van Lanschot JJ, Hulshof MC, van Hagen P, van Berge Henegouwen MI, Wijnhoven BP (2015). Neoadjuvant chemoradiotherapy plus surgery versus surgery alone for oesophageal or junctional cancer (CROSS): long-term results of a randomised controlled trial. Lancet Oncol.

[6] Liu B, Bo Y, Wang K, Liu Y, Tang X, Zhao Y, Zhao E, Yuan L (2017). Concurrent neoadjuvant chemoradiotherapy could improve survival outcomes for patients with esophageal cancer: a meta-analysis based on random clinical trials. Oncotarget.

[7] Wang DB, Zhang X, Han HL, Xu YJ, Sun DQ, Shi ZL (2012). Neoadjuvant chemoradiotherapy could improve survival outcomes for esophageal carcinoma: a meta-analysis. Dig Dis Sci.

[8] Merritt RE, Whyte RI, D'Arcy NT, Hoang CD, Shrager JB (2011). Morbidity and mortality after esophagectomy following neoadjuvant chemoradiation. Ann Thorac Surg.

[9] Kelley ST, Coppola D, Karl RC (2004). Neoadjuvant chemoradiotherapy is not associated with a higher complication rate vs. surgery alone in patients undergoing esophagectomy. J Gastrointest Surg.

[10] Sjoquist KM, Burmeister BH, Smithers BM, Zalcberg JR, Simes RJ, Barbour A (2011). Survival after neoadjuvant chemotherapy or chemoradiotherapy for resectable oesophageal carcinoma: an updated meta-analysis. Lancet Oncol.

[11] Donahue JM, Nichols FC, Li Z, Schomas DA, Allen MS, Cassivi SD (2009). Complete pathologic response after neoadjuvant chemoradiotherapy for esophageal cancer is associated with enhanced survival. Ann Thorac Surg.

[12] Samson P, Robinson C, Bradley J, Lockhart AC, Puri V, Broderick S (2016). Neoadjuvant chemotherapy versus chemoradiation prior to esophagectomy: impact on rate of complete pathologic response and survival in esophageal cancer patients. J Thorac Oncol.

[13] Gao X, Tsai PC, Chuang KH, Pai CP, Hsu PK, Li SH, Lu HI, van Lanschot JJ, Chao YK (2022). Neoadjuvant Carboplatin/Paclitaxel versus 5-Fluorouracil/Cisplatin in Combination with Radiotherapy for Locally Advanced Esophageal Squamous Cell Carcinoma: A Multicenter Comparative Study. Cancers (Basel).

[14] Berger AC, Farma J, Scott WJ, Freedman G, Weiner L, Cheng JD (2005). Complete response to neoadjuvant chemoradiotherapy in esophageal carcinoma is associated with significantly improved survival. J Clin Oncol.

[15] van Hagen P, Hulshof MC, van Lanschot JJ, Steyerberg EW, van Berge Henegouwen MI, Wijnhoven BP (2012). Preoperative chemoradiotherapy for esophageal or junctional cancer. N Engl J Med.

[16] Blom RL, Sosef MN, Nap M, Lammering G, van den Berkmortel F, Hulshof MC (2014). Comparison of two neoadjuvant chemoradiotherapy regimens in patients with potentially curable esophageal carcinoma. Dis Esophagus.

[17] Wong IYH, Lam KO, Zhang RQ, Chan WWL, Wong CLY, Chan FSY (2020). Neoadjuvant chemoradiotherapy using cisplatin and 5-fluorouracil (PF) versus carboplatin and paclitaxel (CROSS regimen) for esophageal squamous cell carcinoma (ESCC): a propensity score-matched study. Ann Surg.

[18] Rice TW, Patil DT, Blackstone EH (2017). 8th edition AJCC/UICC staging of cancers of the esophagus and esophagogastric junction: application to clinical practice. Ann Cardiothorac Surg.

[19] Mandard AM, Dalibard F, Mandard JC, Marnay J, Henry-Amar M, Petiot JF (1994). Pathologic assessment of tumor regression after preoperative chemoradiotherapy of esophageal carcinoma. Clinicopathologic correlations. Cancer.

[20] Becker K, Mueller JD, Schulmacher C, Ott K, Fink U, Busch R (2003). Histomorphology and grading of regression in gastric carcinoma treated with neoadjuvant chemotherapy. Cancer..

[21] Dindo D, Demartines N, Clavien PA (2004). Classification of surgical complications: a new proposal with evaluation in a cohort of 6336 patients and results of a survey. Ann Surg.

[22] Emami B, Lyman J, Brown A, Coia L, Goitein M, Munzenrider JE (1991). Tolerance of normal tissue to therapeutic irradiation. Int J Radiat Oncol Biol Phys.

[23] Marks LB, Yorke ED, Jackson A, Ten Haken RK, Constine LS, Eisbruch A (2010). Use of normal tissue complication probability models in the clinic. Int J Radiat Oncol Biol Phys.

[24] Herskovic A, Martz K, Al-Sarraf M, Leichman L, Brindle J, Vaitkevicius V (1992). Combined chemotherapy and radiotherapy compared with radiotherapy alone in patients with cancer of the esophagus. N Engl J Med.

[25] National Cancer Institute, National Institutes of Health, US Department of Health and Human Services. Common Terminology Criteria for Adverse Events (CTCAE), Version 4.0. NIH publication 09-7473. Published May 29, 2009; Revised June 14, 2010. https://ctep.cancer.gov/protocoldevelopment/electronic_applications/docs/CTCAE_4.03.xlsx. Accessed 16 Jan 2023

[26] Reinstaller T, Adolf D, Lorenz E, Croner RS, Benedix F (2022). Robot-assisted transthoracic hybrid esophagectomy versus open and laparoscopic hybrid esophagectomy: propensity score matched analysis of short-term outcome. Langenbecks Arch Surg.

[27] Mariette C, Dahan L, Mornex F, Maillard E, Thomas PA, Meunier B (2014). Surgery alone versus chemoradiotherapy followed by surgery for stage I and II esophageal cancer: final analysis of randomized controlled phase III trial FFCD 9901. J Clin Oncol.

[28] Markar SR, Bodnar A, Rosales J, Song G, Low DE (2013). The impact of neoadjuvant chemoradiotherapy on perioperative outcomes, tumor pathology, and survival in clinical stage II and III esophageal cancer. Ann Surg Oncol.

[29] Duan XF, Tang P, Yu ZT (2014). Neoadjuvant chemoradiotherapy for resectable esophageal cancer: an in-depth study of randomized controlled trials and literature review. Cancer Biol Med.

[30] Honing J, Smit JK, Muijs CT, Burgerhof JG, de Groot JW, Paardekooper G (2014). A comparison of carboplatin and paclitaxel with cisplatinum and 5-fluorouracil in definitive chemoradiation in esophageal cancer patients. Ann Oncol.

[31] Sanford NN, Catalano PJ, Enzinger PC, King BL, Bueno R, Martin NE (2017). A retrospective comparison of neoadjuvant chemoradiotherapy regimens for locally advanced esophageal cancer. Dis Esophagus.

[32] Munch S, Pigorsch SU, Feith M, Slotta-Huspenina J, Weichert W, Friess H (2017). Comparison of neoadjuvant chemoradiation with carboplatin/ paclitaxel or cisplatin/ 5-fluoruracil in patients with squamous cell carcinoma of the esophagus. Radiat Oncol.

[33] Zhu Y, Zhang W, Li Q, Li Q, Qiu B, Liu H (2017). A phase II randomized controlled trial: definitive concurrent chemoradiotherapy with docetaxel plus cisplatin versus 5-fluorouracil plus cisplatin in patients with oesophageal squamous cell carcinoma. J Cancer.

[34] Zhao Y, Song R, Jia Y, Zhang X, Zhang S, Wu C (2022). Comparison of efficacy and safety of taxanes plus platinum and fluorouracil plus platinum in the first-line treatment of esophageal cancer: a systematic review and meta-analysis. Curr Oncol.

[35] Haisley KR, Hart KD, Nabavizadeh N, Bensch KG, Vaccaro GM, Thomas CR (2017). Neoadjuvant chemoradiotherapy with concurrent cisplatin/5-fluorouracil is associated with increased pathologic complete response and improved survival compared to carboplatin/paclitaxel in patients with locally advanced esophageal cancer. Dis Esophagus.

[36] Worrell SG, Towe CW, A. Dorth J, Machtay M, Perry Y, Linden PA (2020). Higher doses of neoadjuvant radiation for esophageal cancer do not affect the pathologic complete response rate or survival: a propensity-matched analysis. Ann Surg Oncol.

[37] Blum Murphy M, Xiao L, Patel VR, Maru DM, Correa AM, G. Amlashi F (2017). Pathological complete response in patients with esophageal cancer after the trimodality approach: the association with baseline variables and survival-The University of Texas MD Anderson Cancer Center experience. Cancer..

[38] Nehlsen AD, Lehrer EJ, Resende-Salgado L, Rosenzweig KE, Buckstein M (2021). Comparison of pathologic complete response rates and oncologic outcomes in patients with surgically resectable esophageal cancer treated with neoadjuvant chemoradiation to 50.4 Gy vs 41.4 Gy. Cureus..

[39] Wong C, Law S (2017). Predictive factors in the evaluation of treatment response to neoadjuvant chemoradiotherapy in patients with advanced esophageal squamous cell cancer. J Thorac Dis.

[40] Eyck BM, van der Wilk BJ, Noordman BJ, Wijnhoven BPL, Lagarde SM, Hartgrink HH (2021). Updated protocol of the SANO trial: a stepped-wedge cluster randomised trial comparing surgery with active surveillance after neoadjuvant chemoradiotherapy for oesophageal cancer. Trials..

[41] ClinicalTrials.gov Internet. Bethesda (MD): National Library of Medicine (US). 2000 Feb 29 -. Identifier NCT02551458, Comparison of systematic surgery versus surveillance and rescue surgery in operable oesophageal cancer with a complete clinical response to radiochemotherapy (esostrate); 2015 Sep 6. Available from: https://ClinicalTrials.gov/show/NCT02551458. Accessed January 03, 2023.

